# Nurses during war: Profiles‐based risk and protective factors

**DOI:** 10.1111/jnu.13019

**Published:** 2024-08-26

**Authors:** Liat Hamama, Inbal Amit, Michal Itzhaki

**Affiliations:** ^1^ The Bob Shapell School of Social Work Tel Aviv University Tel Aviv Israel; ^2^ Head of Nursing Division Samson Assuta Ashdod Hospital Ashdod Israel; ^3^ Nursing Department, the Stanley Steyer School of Health Professions, Faculty of Medical & Health Science Tel Aviv University Tel Aviv Israel

**Keywords:** adjustment disorder, coping, nurses, psychological distress, reactive protective factors, resilience, resources, wartime

## Abstract

**Introduction:**

Nurses in southern Israel's public hospitals were exposed to unusual traumatic events following the October 7, 2023, Hamas attack on Israel, and the ensuing Swords of Iron War. This study aimed to clarify the complexity of wartime nursing by identifying profiles based on risk factors (i.e., psychological distress and adjustment disorders) and protective factors (i.e., positive affect (PA), resilience, and perceived social support [PSS]).

**Design:**

This study utilizes a cross‐sectional design.

**Method:**

Two hundred nurses at a major public hospital in southern Israel completed self‐report questionnaires. A latent profile analysis (LPA) was conducted to identify distinct profiles based on nurses' risk and protective factors. Differences in profiles were examined alongside sociodemographic and occupational variables and traumatic event exposure. The LPA was conducted using MPlus 8.8 Structural Equation Modeling (SEM) software.

**Findings:**

Two distinct profiles were identified: “reactive” and “resilient.” The “reactive” group included nurses who had higher risk factor scores (psychological distress and adjustment disorder), whereas the “resilient” group included nurses who had higher protective factor scores (PA, resilience, and PSS). Furthermore, nurses in the “reactive” group were younger, with greater seniority, worse self‐rated health, and a higher frequency of kidnapped family members compared to nurses from the “resilient” group.

**Conclusion:**

Nurses in wartime are at risk if identified as “reactive.” Identifying these profiles can assist in developing effective support practices to help nurses cope with wartime challenges and maintain their mental well‐being.

**Clinical Relevance:**

Healthcare organizations should tailor interventions to prepare and support nurses of various ages and experience levels, during and after conflicts. This approach aims to reduce risk factors and promote protective factors among nurses during wartime.

## INTRODUCTION

The practice of nursing in a hospital environment is significantly affected by both acute and cumulative workplace conditions (Ganz et al., 2019). As frontline responders during wartime and other conflicts, nurses play a pivotal role in delivering immediate medical care and support to those injured or impacted (Sberro‐Cohen et al., 2023). Additionally, nurses can experience direct threats to their safety and the safety of their loved ones during wartime (Ben‐Ezra et al., 2011; Sberro‐Cohen et al., 2023). These challenges and conditions might create a confluence of primary and secondary trauma (i.e., direct threat versus being a witness to someone else's trauma), which may affect nurses' mental health (Ben‐Ezra & Bibi, 2016; Sberro‐Cohen et al., 2023). As such, there is a need to focus on nurses' coping strategies and resources (both personal and environmental), which are essential for navigating such difficult times and sustaining optimal mental and psychological health (Ben‐Ezra & Bibi, 2016).

The focus of the current study was on Israeli hospital nurses following an armed conflict between Israel and Hamas‐led Palestinian militant groups that has been raging, primarily in and around the Gaza Strip, since October 7, 2023. The conflict began with a surprise attack by Hamas on southern Israel from the Gaza Strip, dubbed “Operation Al Aqsa Flood.” This operation involved an extensive rocket assault across the nation and the infiltration into Israel by a significant number of terrorists, leading to the deaths of approximately 1300 civilians. Additionally, 252 individuals—including Israelis and foreigners, infants, children, women, and elderly people—were taken captive and held in the Gaza Strip (https://www.gov.il/en/pages/swords‐of‐iron‐war‐in‐the‐south‐7‐oct‐2023). In response, on October 27, 2023, Israel began its own operation—the Swords of Iron War. These chain events are considered traumatic events: “actual or threatened death, serious injury, or sexual violence” (American Psychiatric Association, 2013, p. 271). As such, nurses at public hospitals have been at risk of combined exposure to traumatic events, including events that pose a risk both to their own lives and to those of their family members (Ben‐Ezra & Bibi, 2016; Sberro‐Cohen et al., 2023). In this study, we aimed to identify distinct profiles based on a combination of risk factors (i.e., psychological distress and adjustment disorder) and protective factors (i.e., positive affect [PA], perceived social support [PSS], and resilience). We also sought to explore the differences between these profiles in relation to participants' exposure to threatening events and their background variables (i.e., sociodemographic and occupational).

The transactional theory by Lazarus and Folkman (1984) proposes that the intensity of a stress reaction is influenced by the mediating role of appraisal—a cognitive process through which meaning is ascribed to events. This process stimulates emotions considered threatening, challenging, or harmful, and prompts the use of coping strategies to manage emotions or directly contend with the stressors. These coping efforts lead to an outcome, altering the interaction between the individual and their environment, which is then evaluated as positive, negative, or unresolved (Biggs et al., 2017). A positive resolution of stressors brings about positive emotions, whereas unresolved or negative resolutions cause distress, leading the individual to explore additional coping mechanisms to address the stressor (Biggs et al., 2017). Drawing on the transactional theory, we focused on two negative outcomes: psychological distress and adjustment disorder (risk factors). *Psychological distress* is described as an uncomfortable emotional experience in response to a specific stressor or demand that leads to harm, whether temporary or permanent, to the individual (Ridner, 2004). An *adjustment disorder* represents a maladaptive response to a stressful event, ongoing psychosocial challenges, or a response to a combination of stressful life circumstances. The nature and severity of an adjustment disorder can be shaped by the characteristics and duration of the stressor (e.g., single, repeated, cumulative, or long‐term events), past experiences, and environmental factors (Maercker & Lorenz, 2018).

In addition, we examined the positive resolution of the stressors—*positive affect*—which concerns daily life experiences and includes the experiential evaluation of one's positive emotions, such as satisfaction, happiness, energy, joy, and relaxation (Keyes, 2006). Individuals with high levels of PA exhibit a stronger sense of control over their lives, manage stress more effectively, engage in more active and open thinking, and set personal life goals (Keyes & Ryff, 2000). In addition to PA, we focused on resources as another protective factor. According to Hobfoll's Conservation of Resources (COR) theory (Hobfoll, 1989), stress arises from three scenarios: the actual depletion of resources, the potential risk of losing them, or the inability to obtain resources following a significant investment (Hobfoll, 1989, 2002). According to Hobfoll's (1989) theory, people attempt to acquire, conserve, and protect their resources. In this study, we focused on resilience (personal resource) and PSS (interpersonal resource). *Resilience* represents a personal characteristic: “the personal qualities that enable one to thrive in the face of adversity” (Connor & Davidson, 2003, p. 76). Individuals who demonstrate psychological resilience are likely to exhibit effective coping strategies (Fletcher & Sarkar, 2013). PSS concerns beliefs about the availability of various types of support from one's social networks (Gottlieb & Bergen, 2010), including family (extended or nuclear), friends (individuals who are not family), and significant others (partners or others considered particularly close; Zimet et al., 1988).

Finally, we strove to examine nurses' distinct profiles based on risk and protective factors, alongside background variables (i.e., sociodemographic and occupational), and exposure to various October 7th/Swords of Iron‐related traumatic events (i.e., direct exposure, witnessing the trauma, learning that the trauma happened to a close relative or close friend, indirect exposure to aversive details of the trauma in the course of professional duties; American Psychiatric Association, 2013). Regarding background variables, a previous meta‐analysis (Kisely et al., 2020) of 59 papers on the psychological reactions of healthcare staff during virus outbreaks (e.g., SARS, MERS, Ebola, H1N1, and COVID‐19) revealed that being female, young, and less experienced (in terms of seniority) could render individuals more vulnerable to psychological distress. Conversely, Godifay et al. (2018) demonstrated that Ethiopian healthcare workers (nurses and physicians) who had work experience of ≥5 years had 4.1 times higher odds of developing work‐related stress than those who had ≤5 years of experience.

Given the above, our study could potentially add innovative knowledge to the empirical literature about nurses during wartime, specifically regarding risk and protective factors, offering a clearer picture of the complexity of being a nurse during such periods. Identifying distinct profiles might enable the development of more effective support practices to maintain nurses' mental health.

## METHODS

### Participants

The sample comprised 200 nurses working at a major public hospital in Israel's south, aged 23–66 years (*M* = 42.62, *SD =* 9.17). Of the participants, 182 (91%) were female; 167 (84%) were either married or in a partnership; 175 (88%) identified as Jewish; and 187 (94%) assessed their health as either good or excellent (see Table 1).

**TABLE 1:** Nurses' sociodemographic and occupational characteristics (*n* = 200).

Variables	*N* (%)	Mean (SD)
Gender		
Male	16 (8.0)	
Female	182 (91)	
Other	2 (1.0)	
Marital status		
Married	167 (84)	
Not married	33 (17)	
Education		
Registered nurse (RN) certification without an academic degree	10 (5.0)	
RN with a bachelor's degree	101 (51)	
RN with a master's degree	87 (44)	
RN with a PhD	2 (1.0)	
Religion		
Jewish	175 (88)	
Muslim	6 (3.0)	
Christian	9 (4.5)	
Druze	10 (5.0)	
Religiosity		
Secular	130 (65)	
Traditional	47 (24)	
Religious	18 (9.0)	
Orthodox	5 (2.5)	
Health		
Bad	1 (0.5)	
Not so good	12 (6.0)	
Good	127 (64)	
Excellent	60 (30)	
Role		
Staff nurse	132 (66)	
Nurse in a managerial position	68 (34)	
Years of job seniority		14.34 (10.46)
Years of tenure at the hospital		4.45 (2.17)

In terms of occupational characteristics, 64 nurses (34%) held administrative positions; the range of nursing seniority spanned 3 months to 36 years (*M* = 14.34, *SD* = 10.46); and the average tenure at the hospital was reported as 4.45 years (*SD* = 2.17; Table 1).

Regarding nurses' exposure to October 7th‐related threatening events, 33% (*n* = 39) reported having a family member who was injured in the Hamas attack. Additionally, 7.5% (*n* = 15) reported having a family member who was kidnapped or missing. At the occupational level, 19% (*n* = 38) of the nurses reported treating victims who had been at the Nova Music Festival (i.e., one of the massacre sites at which 364 civilians were killed, many were wounded, and another 40 abducted); 46% (*n* = 91) treated victims from the attacked communities of the Gaza envelope (an area that encompasses the populated areas in the Southern District of Israel that are within 7 km of the Gaza Strip border); 33% (*n* = 66) treated civilians suffering from anxiety; and 25% (*n* = 50) treated wounded soldiers who arrived at the hospital.

### Measures

Participants completed standardized self‐report questionnaires that had previously been employed among Israeli populations and demonstrated robust psychometric properties.

*Background data* included sociodemographic and occupational characteristics. The former comprised details about participants' age, biological sex, marital status, number of children, education level, religion, and religiosity. Additionally, participants' self‐rated health was assessed by a single question: “In general, how do you rate your health?” Responses were given on a scale ranging from 1 (*poor*) to 4 (*excellent*). This metric was previously validated with objective health indicators (Benyamini et al., 2003). Concerning occupational characteristics, participants were asked about their years of nursing experience, tenure at their current workplace, type of position (e.g., full‐time, part‐time), and their role within the workplace, such as being a departmental nurse or holding a managerial position.

*Exposure to threatening events* was assessed through questions that gauged participants' exposure to victims who had undergone various October 7th‐related experiences. This exposure was evaluated within the framework of vulnerability circles, either personal (i.e., having a family member injured/kidnapped) or professional (i.e., Nova Music Festival attendees, residents of the attacked Gaza envelope communities, and civilians suffering from anxiety).

*Adjustment disorder* (ADJ) was assessed using the International Adjustment Disorder Questionnaire (IADQ; Shevlin et al., 2020), divided into three main sections. The first section, the psychosocial stressor checklist, requires responses in a binary format (yes = 1, no = 0). The second section includes a symptom list with three items each for measuring preoccupation symptoms and failure to adapt symptoms—for example, “Since the stressful event(s), I often feel afraid about what might happen in the future”; “Since the stressful event(s), I find it difficult to achieve a state of inner peace.” Responses for these items are provided on a 5‐point Likert scale, ranging from 0 (*not at all*) to 4 (*extremely*). Participants are also asked, “Did these symptoms begin within one month of the stressful event?” with answers given in a binary format (yes = 1, no = 0). The third and final section evaluates functional impairment in social, occupational/educational, and other important domains caused by the symptoms (e.g., “In the past month, have the above symptoms affected any other important part of your life?”), using three items on a 5‐point Likert scale that ranges from 0 (*not at all*) to 4 (*extremely*). In the current study, only the second and third sections—comprising 10 items—were applied. The Hebrew validation for the IADQ was provided by Levin et al. (2022), and the reliability (Cronbach's alpha) for the total score scale was 0.92. In the present study, Cronbach's alpha was 0.80.

*Psychological distress* was assessed by the Kessler Psychological Distress Inventory (K‐6; Kessler et al., 2002) via a six‐item questionnaire “intended to yield a global measure of distress based on questions about anxiety and depressive symptoms” that a person has experienced in the last month (e.g., “How frequently have you felt nervous during the last month?”). Each item is scored on a 5‐point Likert‐type scale from 0 (*none of the time*) to 4 (*all the time*). The total score ranges from 0–24. A cutoff point of 13+ is the optimal cutoff point for assessing the prevalence of serious mental illness, and scores of 19 or higher indicate elevated psychological distress. Cronbach's alpha for the original scale was 0.83 (Kessler et al., 2002). The Hebrew adaptation (Ben‐Ezra & Bibi, 2016) was shown to have a reliability of 0.88, and in the present study, Cronbach's alpha was 0.82.

*Positive affect* was measured by the Positive and Negative Affect Schedule (I‐PANAS‐SF; Thompson, 2007) via five items composing the PA scale (e.g., inspired, attentive). Higher scores on PA items indicate the tendency to experience a good mood. Respondents were requested to rate the statement on a 5‐point scale (*never* to *always*) by comparing themselves during the past 2 weeks with their “usual selves.” The Cronbach's alpha in this study was 0.79.

*Perceived social support* was measured by the Multidimensional Scale of Perceived Social Support (MSPSS; Zimet et al., 1988). The scale consists of 12 items distinguishing PSS from three different sources: family (4 items, e.g., “My family is willing to help me make decisions”); friends (4 items, e.g., “My friends really try to help me”); and a significant other (4 items, e.g., “There is a special person who is around when I am in need”). Participants answered on a 7‐point Likert‐type scale, from 1 (*very strongly disagree*) to 7 (*very strongly agree*). The mean score was calculated; a high score indicated greater levels of PSS. The internal reliability of the original MSPSS for the total scale was *α* = 0.91 (Zimet et al., 1988), and in the current study, it was 0.95.

*Resilience* was measured using the Connor–Davidson Resilience Scale (CD‐RISC 10; Campbell‐Sills & Stein, 2007), a brief version of the CD‐RISC 25 (Connor & Davidson, 2003). Participants were asked to rate the 10 items (e.g., “I believe I can achieve my goals, even if there are obstacles”) on a 5‐point scale, ranging from 0 (*not true at all*) to 4 (*true nearly all the time*), resulting in a possible score range of 0–40. Higher scores on this scale indicate a greater level of resilience. In the current study, Cronbach's alpha was 0.89. [Permission for use of the Hebrew CD‐RISC‐10 was granted by Jonathan Davidson on November 8, 2023.]

### Procedure

The present study was conducted in January–February 2024 (4 weeks) at a general hospital in Israel's south that provides healthcare for about 400,000 civilians as well as for soldiers. The study protocol was approved by the Assuta Ashdod Hospital Institutional Review Board and Helsinki Committee (approval no. 0142‐23‐AAA) and by Tel Aviv University's Institutional Ethics Committee (approval no. 0007542‐1). After approvals, the second author provided, via the hospital's internal email system and WhatsApp, a direct link to an electronic questionnaire run on QUALTRICS. Informed consent was obtained. Participants were informed of the purpose of the study and the eligibility criteria—namely, nurses who could read and speak Hebrew fluently—and they provided their consent electronically (by clicking “I agree to participate”). Respondents took approximately 12 min to complete the questionnaire. Of the 670 potential participants (i.e., all the hospital's nurses), 200 returned the fully completed questionnaires (response rate 29.85%). Thirteen questionnaires were excluded as they were not complete.

### Data analysis

**FIGURE 1:**
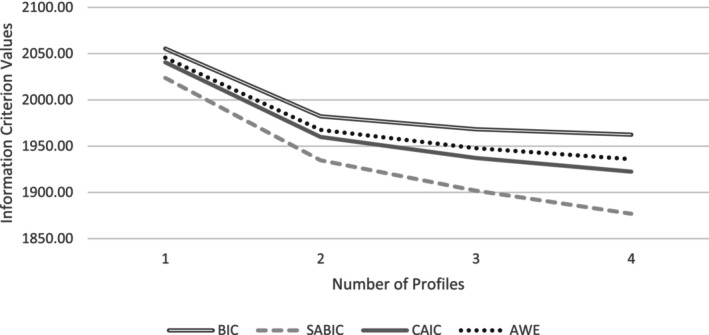
Scree plot of fit indices of the LPA. An elbow is indicated in 2 profiles.

We first applied latent profile analysis (LPA) to estimate distinct profiles in nurses' psychological distress, adjustment disorder, PA, PSS, and resilience (risk and protective factors). To do so, we followed the guidelines of Nylund‐Gibson and Choi (2018) using MPlus 8.8 (Muthén & Muthén, 2023) Structural Equation Modeling (SEM) software. We examined one to four possible profiles using unconditional LPA. To decide on the number of profiles, we used the following information (summarized in Table 1): (i) information criteria (IC)—including the Bayesian Information Criterion (BIC), sample‐size adjusted Bayesian Information Criterion (SABIC), Consistent Akaike Information Criterion (CAIC), and Approximate Weight of Evidence Criterion (AWE)—which are approximate fit indices where lower values indicate superior fit. These ICs were also plotted (see Figure 1) to inspect for an “elbow” point of “diminishing returns” in model fit (equivalent to a scree plot in factor analysis); (ii) we also used likelihood‐based tests—the Vuong–Lo–Mendell–Rubin adjusted likelihood ratio test (VLMR‐LRT) and the bootstrapped likelihood ratio test (BLRT)—which provide *p*‐values assessing whether adding a class leads to a statistically significant improvement in model fit. The BLRT has been shown to be one of the most robust methods across a diversity of modeling conditions (Nylund et al., 2007); and (iii) finally, we employed the Bayes factor (BF) indices used as a pairwise comparison of fit between two neighboring class models with values >10 suggesting “strong” support for the more complex model, and the correct model probability (cmP) that provides an estimate of each model being “correct” out of all models considered. We also considered how the selected models related to each other (e.g., theoretically different) as well as the relative sizes of the emergent profiles. Here, we decided on a minimum profile size of 20 participants. Overall, 200 participants were profiled in this phase. Missing data were handled with Multiple Imputation (Rubin, 2009).

Upon deciding on the ideal number of profiles, we identified and examined the consequences of latent profile membership using distal variables. To do that, we employed the Bolck, Croon, and Hagenaars (BCH) method (Bakk & Kuha, 2021; Bolck et al., 2004). Specifically, we separated the profile enumeration step from subsequent structural analyses, such that profiles were enumerated solely with the chosen latent class indicators measuring the substantive domain of interest (psychological distress, adjustment disorder, PA, PSS, and resilience). Using the selected profile solution, BCH weights were saved alongside the distal variables—treating civilians with anxiety (yes, no), Nova survivors (yes, no), and Gaza envelope survivors (yes, no); having a family member injured (yes, no) or kidnapped (yes, no); age; number of children; religiosity; self‐rated health; and nursing seniority in years. In the 3rd step, the measurement parameters of the latent classes were held fixed while accounting for classification error. Distal variables were included, and their relation to the latent class variable was estimated.

## RESULTS

The pattern of associations between LPA measures (psychological distress, adjustment disorder, PA, PSS, and resilience) and exposure measures is presented in Figure S1, and between LPA measures and background measures in Figure S2.

### Latent profile analysis

**FIGURE 2:**
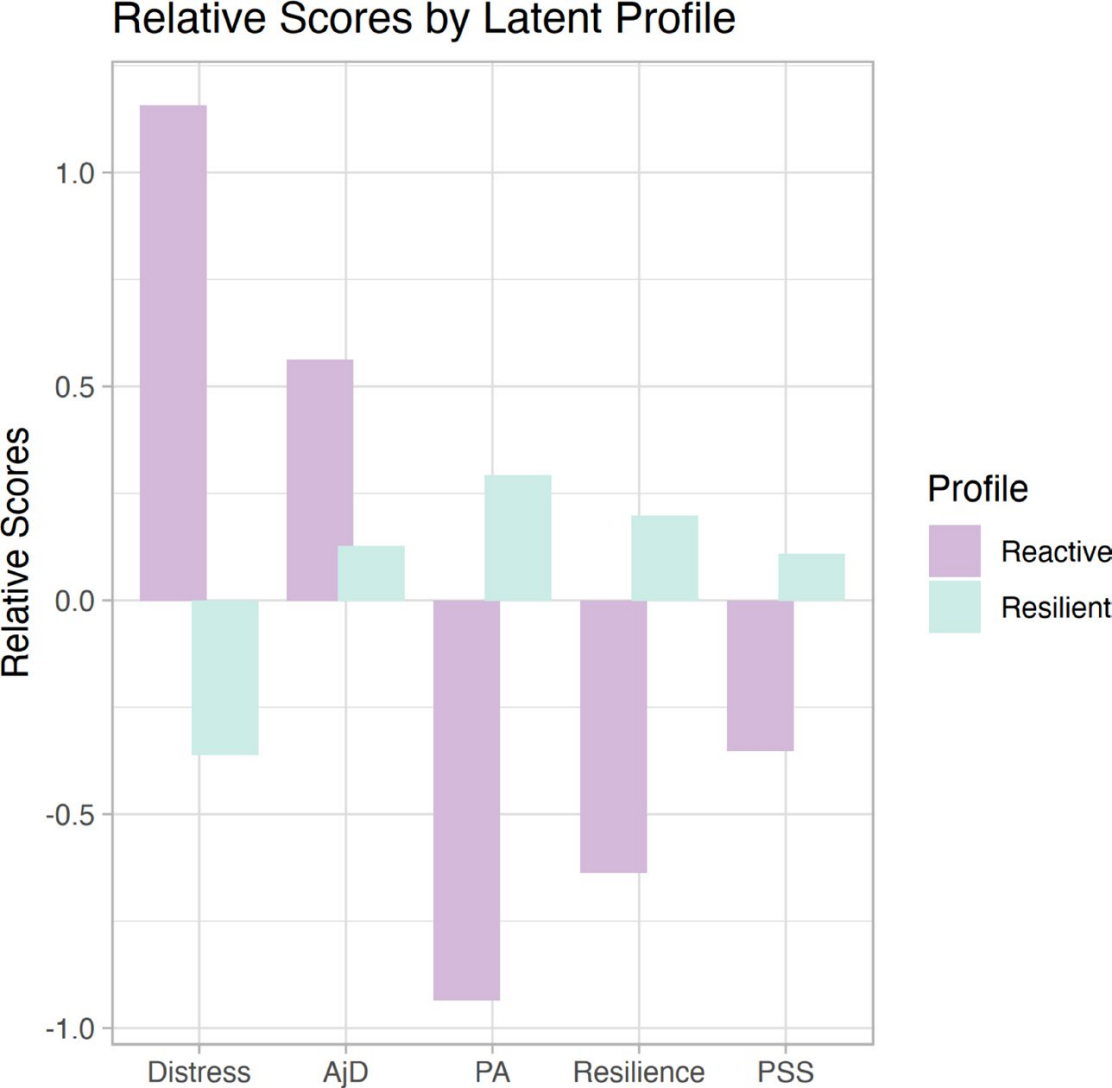
The relative scores of the variables comprise the two profiles. ADJ, adjustment disorder; Distress, psychological distress; PA, positive affect; PSS, perceived social support.

Results are summarized in Table 2. Fit indices did not converge on a single solution, which is generally the rule rather than the exception in applied practice (Nylund‐Gibson & Choi, 2018). The ICs and *cmP* suggested a 4‐profile solution, whereas the likelihood tests and BF supported a 2‐profile solution. In addition, as plotted in Figure 1, an elbow in the ICs was already observed at the 2‐profile solution. Given that the likelihood tests have been shown to be robust across a diversity of modeling conditions (Nylund et al., 2007), we selected the 2‐profile solution in Step 1. The entropy score of 0.74 and the average posterior probabilities (AvePP) scores >0.80 reflect well‐separated profiles (Nagin, 2005). The profiles are presented in Figure 2, comprising “reactive” (*n* = 44) and “resilient” (*n* = 156). The reactive group had significantly higher psychological distress scores and frequency of adjustment disorders than did the resilient group. The reactive group also had lower PA, resilience, and PSS.

**TABLE 2:** Fit indices of LPA.

	1 profile	2 profiles	3 profiles	4 profiles
BIC	2055.44	1982.22	1968.30	**1962.43**
SABIC	2023.76	1934.70	1901.77	**1876.89**
CAIC	2040.63	1960.00	1937.19	**1922.43**
AWE	2045.63	1967.50	1947.69	**1935.93**
VLMR‐LRT		**91.19 (0.015)**	44.31 (0.302)	36.51 (0.651)
BLRT		**94.06 (<0.0001)**	45.71 (0.292)	37.66 (0.592)
BF		**38.91**	2.01	1.34
cmP	0.00	0.17	0.35	**0.47**
Entropy	1.00	0.74	0.77	0.83
LL	−1001.23	−951.37	−928.52	−909.69
% smallest n		0.22	0.12	0.08

*Note*: The best scores in each category are marked in bold.

### Differences between profiles in distal variables

In the third step of the model, significant differences between the profiles were found in exposure to threatening events (specifically, a family member was kidnapped on October 7) and background variables (age, self‐rated health, and seniority). Intercepts and residual standard deviations are presented in Table 3. We found that the “reactive” group had a significantly greater frequency of kidnapped family members (18% vs. 4%), younger age, greater job seniority, and worse self‐rated health as compared with the “resilient” group. The frequency of treating civilians with anxiety, Nova survivors, Gaza envelope survivors, and/or having a family member injured was not related to the differences between the “reactive” and “resilient” groups.

**TABLE 3:** Differences between profiles in the distal variables.

	Reactive		Resilient		*p*
	M/%	SD	M/%	SD	
Took care of anxiety patients (yes)	0.34		0.33		0.862
Took care of Nova survivors (yes)	0.23		0.18		0.496
Took care of Gaza envelope survivors (yes)	0.45		0.46		0.995
Family member injured (yes)	0.47		0.36		0.291
Family member kidnapped (yes)	0.18		0.04		**0.043**
Age	38.98	8.58	43.75	9.02	**0.011**
Number of children	2.99	1.38	3.43	1.21	0.116
Religiosity	1.50	0.85	1.49	0.73	0.927
Self‐rated health	3.01	0.69	3.30	0.51	**0.031**
Job seniority	28.30	13.70	22.42	14.42	**0.048**

*Note*: Significant differences are marked in bold.

## DISCUSSION

This study is the first study in which distinct profiles based on risk factors (psychological distress and adjustment disorder) and protective factors (PA, resilience, and PSS) among nurses in wartime were examined. The findings offer a clear picture of the way nurses have coped in the aftermath of October 7, 2023, terrorist attack on Israeli civilians and during the ongoing Swords of Iron War. Based on LPA, two distinct profiles were revealed: “reactive” and “resilient.” The reactive group consisted of nurses who had higher scores of psychological distress and adjustment disorder and lower scores of PA, resilience, and PSS. Although we attempted to identify relevant studies for each group, the singular characteristics of the October 7th attack and the ensuing Swords of Iron War made it difficult to rely on previous research. Furthermore, studies addressing the consequences of a sequence of traumatic events (Katsoty et al., 2024; Levi‐Belz et al., 2024) have focused on civilian populations rather than on “first responders,” such as nurses.

As stated above, nurses in the “reactive” group were characterized by high scores of psychological distress and frequency of adjustment disorder. Studies have demonstrated a high prevalence of psychological distress among nurses (Belay et al., 2021) in general and particularly during wartime (Ben‐Ezra et al., 2011). Adjustment disorder, for its part, has not yet been examined in the arena of nursing (as per the ICD‐11, adjustment disorder is a stress response syndrome with core symptoms of preoccupations and failure to adapt to the stressor; Shevlin et al., 2020). In this context, a recent study (Ring et al., 2018) on mortality salience after the Sarona terror attack (in Tel Aviv, Israel) revealed that mortality salience served as a predictor associated with adjustment disorder. Karatzias et al. (2021) suggested that there might be a high degree of comorbidity between the latent structures of adjustment disorder and post‐traumatic stress disorder (PTSD), while also noting that their symptom profiles were unique and distinct. Going forward, researchers should consider exploring the latent structure of adjustment disorder, particularly among nurses exposed to war‐related stress. Indeed, nurses are highly susceptible to psychological distress and adjustment disorder due to the nature of their work, which involves witnessing patients' suffering and death, meeting demanding job expectations, managing progressively heavier workloads, and adjusting to evolving work settings, while at the same time receiving minimal professional support (Hsiao‐Yean, 2022). Likewise, Kenny and Hull (2008) concluded that providing care to patients who have sustained multiple traumas and injuries (e.g., soldiers) presents significant challenges. They noted that these injuries create stress not only for the patients and their families but also for the nurses responsible for their care.

A possible explanation for the two risk factors identified in the “reactive” profile can be explained by Terror Management Theory (TMT; Greenberg et al., 1997). Specifically, TMT posits that humanity has developed a range of coping mechanisms to fend off thoughts related to death and to support daily functioning. Consequently, the awareness of our own mortality triggers extreme stress levels, which may lead to overwhelming anxiety. This potential anxiety can be mitigated by adhering to cultural worldviews, maintaining self‐esteem, and fostering close relationships—referred to as anxiety‐buffering systems (Pyszczynski et al., 2015). In this scenario, nurses exposed to various October 7th‐related incidents may have perceived their anxiety‐buffering systems (i.e., worldview, self‐esteem, and relationships) as having been disrupted and actively threatened. Their thoughts of death became a central focus and attempts to entirely erase these thoughts from their minds proved to be futile. As such, their vulnerability to psychological disorders, such as psychological distress and adjustment disorder, increased.

Furthermore, the “reactive” group profile included lower PA, resilience, and PSS. Concerning PA, Palgi et al. (2012) reported that hospital personnel (nurses and physicians) with a positive congruent (high PA and low negative affect) had a lower level of peritraumatic symptoms during exposure to the Second Lebanon War (2006) and Israel's Operation Cast Lead (2008–2009). According to Fredrickson's (1998) broaden‐and‐build theory, positive emotions temporarily expand individuals' attention and thinking, allowing them to access higher‐level connections and a broader range of perceptions or ideas than usual. These expanded perspectives enable individuals to identify and develop significant personal resources, become better equipped to manage stressful situations, think in a more active and open manner, and effectively seize opportunities (Keyes & Ryff, 2000).

Resilience (i.e., “the personal qualities that enable one to thrive in the face of adversity”; Connor & Davidson, 2003 p. 76) and PSS (from family, friends, and significant others) have both been reported to protect nurses from stress by mitigating or counterbalancing the negative effects of the stressful events they encounter (e.g., Henshall et al., 2020; Sberro‐Cohen et al., 2023). Hobfoll's COR theory (Hobfoll, 1989) underscores the importance of personal, social, and material resources that individuals strive to acquire, maintain, and protect in times of stress and adversity. In this context, resilience and PSS can be seen as resources that may have enabled the nurses to cope with the heightened October 7th‐related demands they faced. Thus, possessing these resources (protective factors) may be linked to acquiring additional resources that are beneficial in tackling stressful situations, whereas a lack of resources may be linked to being vulnerable to resource loss, with initial losses potentially leading to further losses (Hobfoll, 2002). In line with COR theory, it might be that the hospital nurses categorized in the “reactive” group may have failed to acquire resources and, as a result, were more vulnerable to psychological distress and adjustment disorder.

The study findings regarding the differences between the profiles in distal variables revealed that the “reactive” group (i.e., nurses who reported higher scores of psychological distress and adjustment disorder, and lower scores of PA, resilience, and PSS) were younger, had greater nursing seniority, had worse self‐rated health, and had a higher frequency of kidnapped family members. Regarding seniority, as mentioned earlier, previous studies (Belay et al., 2021; Godifay et al., 2018) have shown that nurses with more work experience face higher odds of developing work‐related stress and psychological distress compared to those with fewer years of experience. Furthermore, concerning age, research on older adults during stressful events has shown that they develop psychological resilience by utilizing coping strategies gained from past life challenges and experiences (Lind et al., 2021). A possible explanation for the differences in nurses' age and seniority as related to the two groups (reactive/resilient) may be attributed to the nature of nursing work in wartime, the workplace environment, the social context, and the occupational hazards encountered during military conflicts (Fink & Milbrath, 2023). Specifically, younger nurses might not yet have a comprehensive understanding of the nursing profession's complexities and may lack the psychological robustness needed to handle the various challenges associated with their roles. Conversely, more experienced nurses often encounter increased stress due to their supervisory duties and their more frequent interactions with injured patients, potentially amplifying their psychological distress.

Regarding the differences in self‐rated health among the nurses in the two groups, Martin et al. (2022) found that a better‐rated health status was a major predictor of being more self‐compassionate, happier, more satisfied with life, and less stressed among undergraduate nursing students. Furthermore, worse self‐rated health was found to be associated with negative psychosocial job characteristics (i.e., inadequate social support and low sense of coherence) among Lithuanian hospital nurses (Malinauskiene et al., 2011). It thus appears that these background variables (age, nursing seniority, and self‐rated health) may have acted as antecedents in the “reactive” group profile and contributed to their vulnerability.

Lastly, a significantly higher frequency of exposure to threatening events, especially having kidnapped family members, was found in the “reactive” group profile. Nurses routinely encounter a variety of potentially psychologically traumatic events as part of their daily work activities, and cumulative exposure to these potentially traumatic events can lead to clinically significant symptoms of mental disorders, including PTSD, depression, anxiety, and panic disorder symptoms (Stelnicki et al., 2021). However, on and post‐October 7th, nurses in Israel came face to face with the largest mass casualty incident and the largest terror attack in Israel's history (Codish et al., 2024). They were exposed to events that directly threatened them, including the kidnapping of their own relatives, while treating and caring for wounded civilians and soldiers. In this vein, it appears that the nurses in the “reactive” group were subjected to cumulative stressors, including the traumatic events of their daily work, as well as the October 7th‐related events that directly and indirectly threatened them. Researchers in the field should continue to explore how life‐threatening events directly experienced by, witnessed by, or learned about by nurses in their occupational practice and personal lives are related to the “reactive” group profile.

### Limitations

Several study limitations should be noted. First, the cross‐sectional approach offers a limited snapshot of the long‐term effects of trauma, not accounting for possible recovery or delayed symptoms. Nevertheless, it should be mentioned that the checklist for reporting cross‐sectional studies, based on the STROBE (Strengthening the Reporting of Observational Studies in Epidemiology) guidelines, provides a comprehensive framework to ensure clarity, transparency, and completeness in reporting (Von Elm et al., 2014). Second, the “reactive” group was relatively small and was identified at a single public hospital in southern Israel, limiting the study's broader applicability. Third, this study relied on self‐reported data. Self‐reporting can be limited by issues, such as memory bias, reporting bias, common method variance, and social desirability bias (Paulhus & Vazire, 2007). Furthermore, it is possible that self‐report questionnaires do not fully capture internal experiences, as well as excluding potential pre‐existing risk factors or the additional stress of war‐related occupational, economic, and personal challenges. Fourth, we did not examine differences in research variables between nurses working in acute wards (such as intensive care and operating rooms), chronic wards (e.g., rehabilitation departments), or outpatient clinics. Future research should include in‐depth interviews to explore risk and protective factors more comprehensively, and longitudinal studies to uncover factors affecting nurses' psychological well‐being amid ongoing trauma (e.g., the Israel–Hamas conflict). Despite these limitations, the findings highlight the importance of identifying at‐risk nurses facing cumulative stress. Furthermore, these findings—which suggest that researchers should focus on risk and protective factors for those in high‐stress frontline roles—can be generalized, especially to conflict zones.

### Implications for health policy

During wartime, hospital nurses face significant challenges. Healthcare organizations need to develop strategies to prepare and support nurses of various ages and levels of experience, especially targeting younger nurses or those with longer tenures who have been identified as susceptible to psychological distress and adjustment disorders. Implementing programs that boost nurses' resilience during and after conflicts could reduce the risks of post‐traumatic stress, burnout, and workforce turnover. Segev (2023) highlighted the importance of emotional and professional support for critical care nurses in wartime to help them process traumatic experiences and develop effective coping mechanisms. Moreover, it is crucial for policymakers to officially recognize nurses' contributions during wartime and document their experiences in professional journals and public media. Such recognition not only honors their service but also offers valuable lessons for handling future crises.

## CONCLUSIONS

Following the events of October 7, 2023, and the ensuing Swords of Iron War in Israel, this study is the first study in which distinct profiles were investigated, based on risk and protective factors, among nurses working at a public hospital in southern Israel. These nurses encountered highly unusual incidents in their personal lives (e.g., having a family member injured, kidnapped, or otherwise unaccounted for post‐October 7th) and in their professional role (e.g., treating injured victims from the Nova Music Festival and from the communities that were attacked, civilians suffering from anxiety attacks, and wounded soldiers upon their arrival at the hospital). We identified two distinct profiles—“reactive” and “resilient”—differentiated by levels of psychological distress, adjustment disorder, PA, resilience, and PSS. Additionally, nurses in the “reactive” group exhibited a higher frequency of having kidnapped family members, poorer self‐rated health, younger age, and greater nursing seniority. Identifying these profiles can aid in developing effective support practices to help nurses cope with wartime challenges and maintain their mental well‐being. Future studies should consider a comparative analysis of nurses' coping mechanisms and challenges during wartime across different countries. Such research would provide a broader understanding of the varied experiences and strategies nurses employ globally, offering valuable insights and enhancing the overall relevance and applicability of the findings.

## FUNDING INFORMATION

This research received no specific grant from any funding agency.

## CONFLICT OF INTEREST STATEMENT

The authors declare no potential conflicts of interest concerning the research, authorship, and/or publication of this study.

## IRB STATEMENT

The study protocol was approved by the Assuta Ashdod Hospital Institutional Review Board and Helsinki Committee (approval no. 0142‐23‐AAA) and by Tel Aviv University's Institutional Ethics Committee (approval no. 0007542‐1).

## CLINICAL RESOURCES

- The Israel Trauma Coalition: https://israeltraumacoalition.org/en/.
- International Council of Nurses Board New Statement on Israel‐Gaza Conflict: https://www.icn.ch/news/international‐council‐nurses‐board‐new‐statement‐israel‐gaza‐conflict.
- ANA Nursing Resources Hub—Three Steps to Build Resilience: https://www.nursingworld.org/content‐hub/resources/nursing‐leadership/three‐steps‐to‐build‐resilience/.

## Supporting information


Data S1.

## Data Availability

The data that support the findings of this study are available from the corresponding author upon reasonable request.
