# Supplementary material for: Nurses during war: Profiles‐based risk and protective factors
Source: J Nurs Scholarsh. 2024 Aug 26;57(2):228–38. doi: 10.1111/jnu.13019 (PMC11931983; doi:10.1111/jnu.13019)
Supplement: Supplementary file 1 — Data S1. [file JNU-57-228-s001.docx]

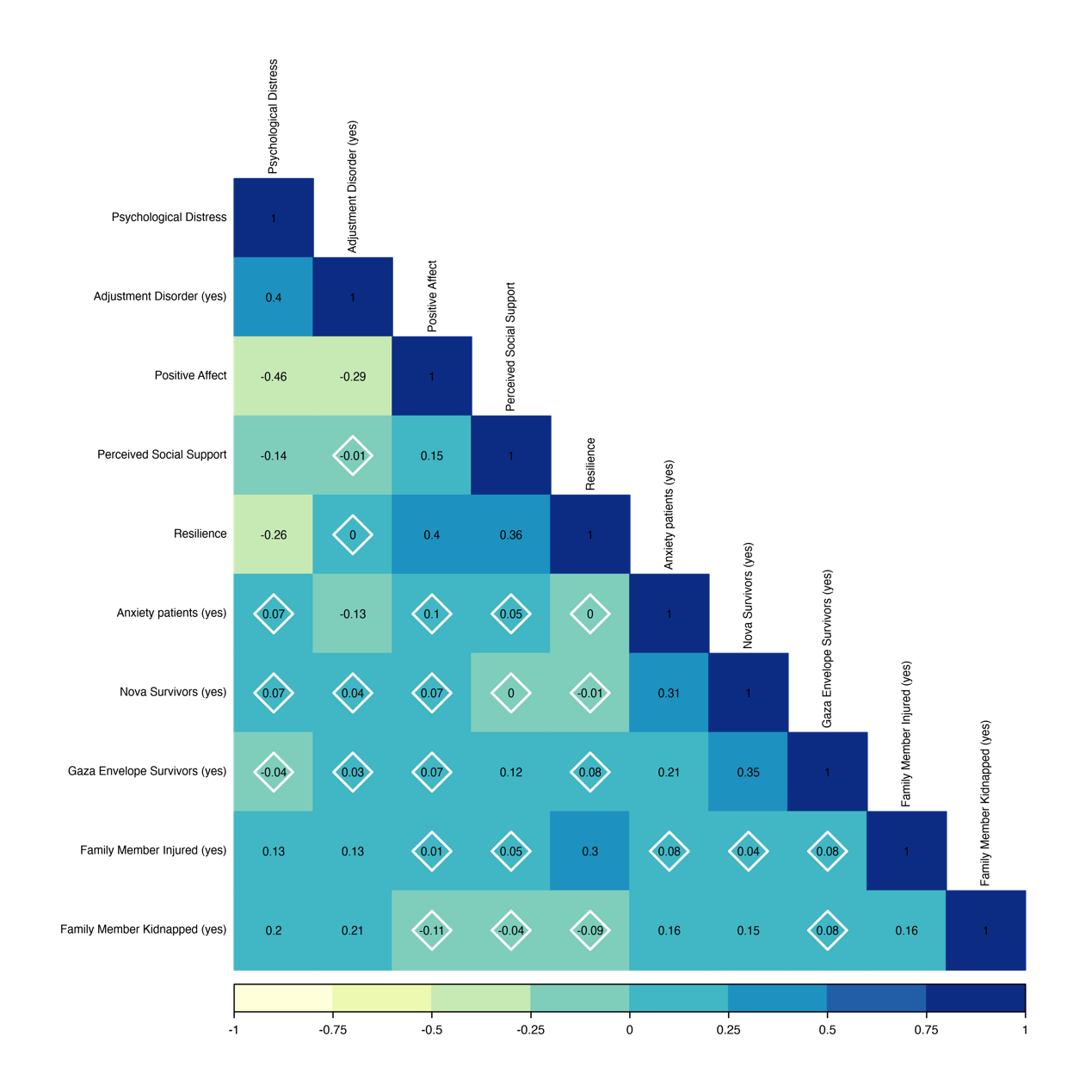


*Supplementary Figure 1*. The pattern of associations between LPA measures (psychological distress, adjustment disorder, positive affect, perceived social support, and resilience) and exposure measures.

Note: Non-significant values are marked with a white diamond; Other values are significant at *p* < .05.


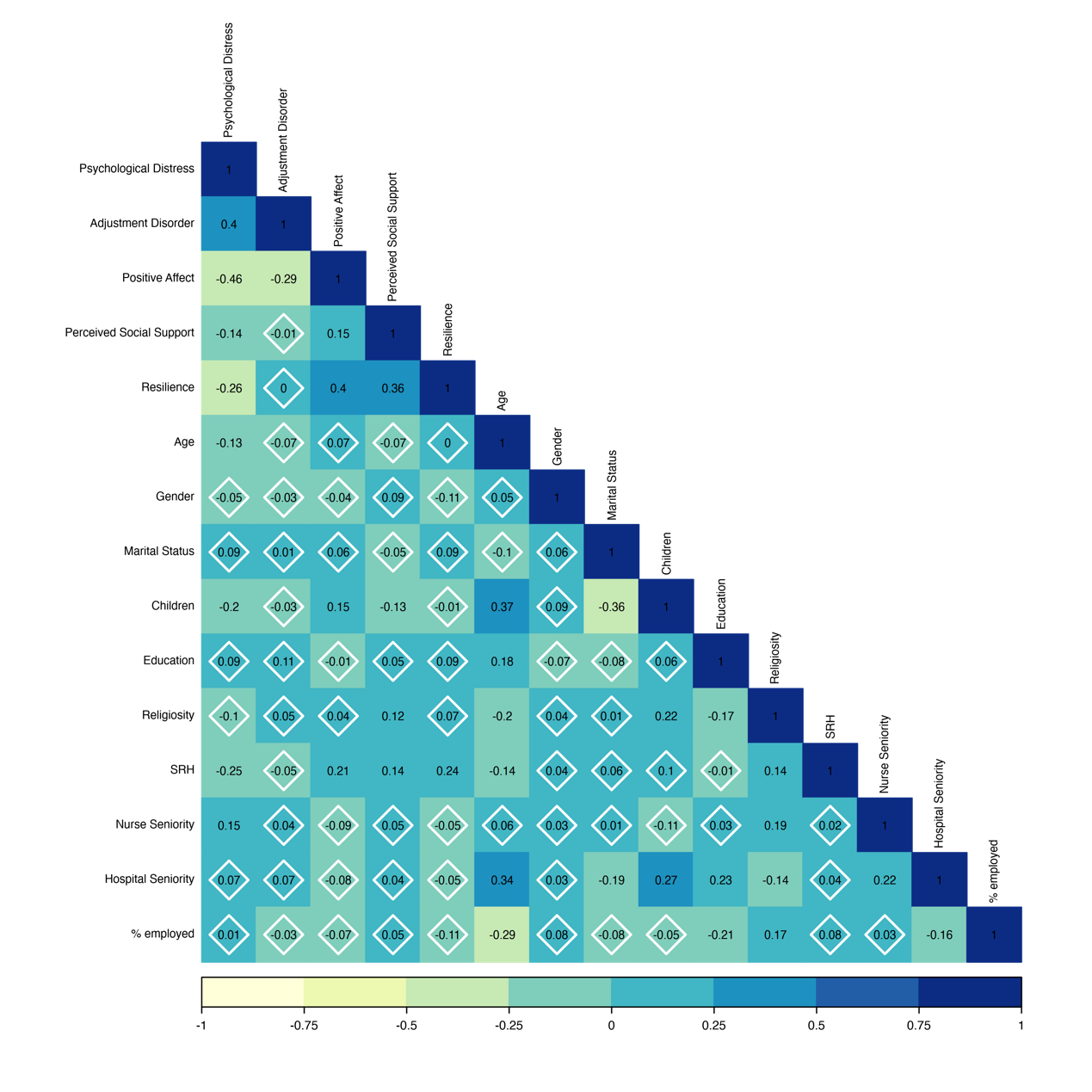
 *Supplementary Figure 2*. The pattern of associations between LPA (psychological distress, adjustment disorder, positive affect, perceived social support, and resilience) and background measures (age, gender, marital status, children, education, religiosity, SRH = self-rated health, nurse seniority, hospital seniority, % employed)

Note: Non-significant values are marked with a white diamond; Other values are significant at *p* < .05.
